# Comparative Multi-Omics Analysis Identifies Shared Transcriptomic Signatures and Therapeutic Targets in Alzheimer’s, Parkinson’s, and Huntington’s Diseases

**DOI:** 10.3390/cimb47120976

**Published:** 2025-11-24

**Authors:** Luai Ibrahim Alharbi, Elsayed Badr, Abdallah Donia, Eman Monir

**Affiliations:** 1 Department of Science and Artificial Intelligence, Faculty of Information Technology, Monash University, Clayton, VIC 3800, Australia; 2 Department of Information Systems, College of Information Technology, Misr University for Science and Technology (MUST), Giza P.O. Box 77, Egypt; 3 Department of Scientific Computing, Faculty of Computer and Artificial Intelligence, Benha University, Benha 13518, Egypt; 4 The Egyptian School of Data Science (ESDS), Benha, Egypt

**Keywords:** Alzheimer’s disease, Parkinson’s disease, Huntington’s disease, differential gene expression, RNA-seq, gene ontology, protein–protein interaction network, neuroinflammation

## Abstract

Alzheimer’s disease (AD), Parkinson’s disease (PD), and Huntington’s disease (HD) are major neurodegenerative disorders that share certain pathological features but differ in their genetic etiology and clinical presentation. Their potential molecular intersections remain incompletely understood. In this research, we conducted a comparative transcriptomic analysis using postmortem brain RNA-seq datasets from AD (GSE53697), PD (GSE68719), and HD (GSE64810) to identify shared and disease-specific transcriptional signatures. Differentially expressed genes (DEGs) were determined and functionally characterized through Gene Ontology (GO) enrichment. Protein–protein interaction (PPI) networks were generated using STRING and visualized in Cytoscape to identify central hub genes, followed by gene–disease and drug-interaction analyses to assess functional and therapeutic relevance. Ten DEGs were found to overlap among the three disorders, exhibiting variable directions of regulation across diseases. Enrichment analysis indicated convergence on immune- and inflammation-related biological processes. Key hub genes, including *MMP9*, *LCN2*, *CXCL2*, *CCL2*, *S100A8*, and *S100A9*, were identified as central nodes within the PPI network. Although the overlap in DEGs was limited, the findings suggest that neuroinflammatory signaling represents a shared molecular theme across AD, PD, and HD, warranting further validation in independent cohorts.

## 1. Introduction

### 1.1. Background and Motivation

Alzheimer’s disease (AD), Parkinson’s disease (PD), and Huntington’s disease (HD) are progressive neurodegenerative disorders characterized by the gradual loss of specific neuronal populations, leading to cognitive, behavioral, and motor impairments. AD represents the most prevalent form of dementia, primarily affecting memory and cognitive functions in the elderly population [1,2,3]. Age remains the strongest risk factor for AD [4,5], and the disease is defined neuropathologically by the presence of extracellular amyloid-β plaques and intracellular neurofibrillary tangles composed of hyperphosphorylated tau protein [6,7]. These pathological features contribute to synaptic dysfunction, neuronal loss, and widespread brain atrophy.

HD is a rare autosomal dominant disorder caused by an expanded cytosine–adenine–guanine (CAG) trinucleotide repeat in the HTT gene, leading to the production of a mutant huntingtin protein with a polyglutamine tract [8,9,10]. The resultant protein aggregation causes neuronal toxicity, particularly in the striatum and cortex, producing characteristic motor abnormalities, psychiatric symptoms, and cognitive decline [10].

PD is the second most common neurodegenerative disorder after AD [11]. It manifests with bradykinesia, rigidity, resting tremor, and postural instability due to the progressive degeneration of dopaminergic neurons in the substantia nigra pars compacta. PD prevalence increases with age, affecting approximately 1% of individuals over 60 years [12,13], and is expected to rise by more than 30% by 2030 due to global population aging [14].

Although AD, PD, and HD are clinically distinct, accumulating evidence suggests they share convergent molecular mechanisms. All three involve protein misfolding and aggregation, mitochondrial dysfunction, oxidative stress, and neuroinflammatory responses [15,16]. Aberrant protein interactions at endoplasmic reticulum–mitochondria contact sites have been implicated in neuronal vulnerability across these disorders [15]. Moreover, disrupted calcium homeostasis and immune activation, particularly microglial and macrophage-mediated synaptic pruning, have been observed in both AD and HD [16,17]. Such findings highlight the possibility of overlapping pathogenic processes that could represent unified therapeutic targets.

To explore these relationships, the present study performed a comparative transcriptomic analysis using three publicly available postmortem RNA-seq datasets: PD (GSE68719) [18], HD (GSE64810) [19], and AD (GSE53697) [20]. Differentially expressed genes (DEGs) were identified for each disease, followed by the determination of shared DEGs across all three. These common DEGs were subsequently analyzed through the following:

- Functional enrichment and pathway analysis to identify shared biological processes.
- Construction of protein–protein interaction (PPI) networks and hub gene identification.
- Gene–disease association analysis to contextualize shared genes within known disease frameworks.
- Protein–drug interaction analysis to identify potential therapeutic targets.

Despite strong evidence of clinical and pathological overlap among AD, PD, and HD, the molecular mechanisms linking them remain poorly defined. Comparative bioinformatics studies that systematically examine these diseases together are still limited. Therefore, this study aims to elucidate the shared transcriptomic signatures and molecular pathways underlying these disorders through an integrated, multi-step bioinformatics workflow.

### 1.2. State of the Art

Previous research has addressed shared genetic or molecular features among neurodegenerative disorders, but often had limited scope.

Wainberg et al. [21] conducted a cross-disorder genome-wide association meta-analysis and identified eleven genetic risk loci shared among AD, PD, and amyotrophic lateral sclerosis (ALS), implicating lysosomal dysfunction, neuroinflammation, oxidative stress, and DNA damage response pathways. However, this study did not assess shared DEGs or transcriptomic enrichment across AD, HD, and PD.

García-Marín et al. [22] examined genetic correlations between PD and subcortical brain structures through a meta-analysis of GWAS data from over 1.4 million individuals, revealing a shared genetic architecture between PD and specific brain regions, such as the putamen and caudate nucleus. Pathway analysis implicated mitophagy and vesicle trafficking, but the study excluded AD and HD.

Wang et al. [23] compared PD and major depressive disorder (MDD) using RNA-seq datasets and identified 45 shared DEGs, highlighting immune cell infiltration as a common mechanism. Although informative, this study focused on psychiatric comorbidity rather than inter-neurodegenerative comparisons.

Shim et al. [24] compared AD and cerebral adrenoleukodystrophy (cALD) through gene set enrichment and WGCNA, revealing overlapping inflammatory and apoptotic signaling pathways, yet again did not include PD or HD.

Termine et al. [25] applied a hybrid network and machine learning approach to PD transcriptomic data, identifying two molecular subtypes with distinct regulatory networks, while Li et al. [26] integrated genetic evidence to prioritize 124 PD-associated genes. Similarly, Dai et al. [27] developed a clinical genetic risk model to predict AD-related neuropathology in PD and dementia with Lewy bodies, demonstrating genetic overlap but not shared gene expression analysis.

Collectively, these studies reveal partial overlaps among neurodegenerative diseases but lack an integrative transcriptomic comparison encompassing AD, PD, and HD.

### 1.3. Aim of This Research

Few studies have systematically investigated the shared transcriptional mechanisms underlying AD, HD, and PD. Most prior analyses have been disease-specific, lacking comparative enrichment, PPI, or gene–disease integration across all three disorders.

The objectives of this study are therefore as follows:

- Identify DEGs for AD, PD, and HD using RNA-seq datasets (GSE53697, GSE68719, and GSE64810).
- Determine overlapping DEGs shared among the three diseases.
- Perform functional enrichment to identify shared pathways and ontologies.
- Construct PPI networks, prioritize hub genes, and explore their biological significance using Cytoscape (version 3.10.3) and CytoHubba (https://apps.cytoscape.org/apps/cytohubba accessed on 15 November 2024).
- Analyze gene–disease and protein–drug interactions to link transcriptomic patterns to functional and therapeutic contexts.
- Present a comprehensive workflow integrating these analyses to provide a unified view of molecular convergence among AD, PD, and HD.

**Figure 1:**
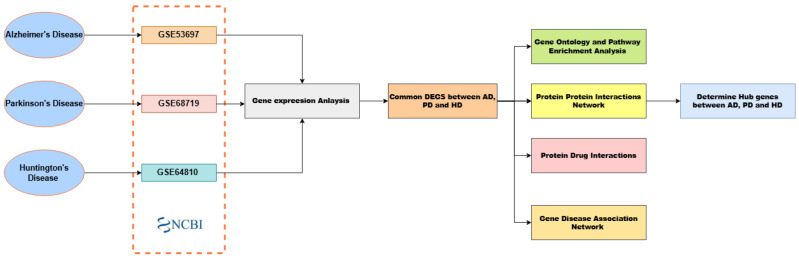
Overview of the workflow illustrating RNA-seq data processing, DEG analysis, enrichment, PPI network construction, and hub gene identification across AD, PD, and HD.

The proposed framework (Figure 1) outlines the sequential workflow of the study, beginning with RNA-seq data acquisition from NCBI GEO, DEG identification, GO and pathway enrichment, PPI construction, hub gene selection, protein–drug interaction mapping, and gene–disease network integration.

The remainder of the paper is organized as follows: Section 2 details the materials and methods, Section 3 presents the results, Section 4 discusses the findings, and Section 5 concludes the study.

## 2. Materials and Methods

### 2.1. Datasets

The Gene Expression Omnibus (GEO) database, maintained by the National Center for Biotechnology Information (NCBI) [28], was used to explore the shared transcriptomic landscape among Alzheimer’s disease (AD), Parkinson’s disease (PD), and Huntington’s disease (HD). Publicly available RNA-Seq datasets were selected based on clear diagnostic criteria, sufficient sample size, and comparable tissue origin to minimize confounding variability.

The following datasets were analyzed:

- AD (GSE53697): This dataset includes 9 control and 8 AD postmortem human brain samples from the Brodmann area 9 (BA9) region. Sequencing was performed using the Illumina HiSeq platform for Homo sapiens [29].
- PD (GSE68719): Contains 44 neurologically healthy and 29 PD postmortem BA9 samples. Differential expression for this dataset was initially assessed using the GEO2R tool (https://www.ncbi.nlm.nih.gov/geo/geo2r/; accessed on 15 August 2024) [30].
- HD (GSE64810): Contributed by Labadorf et al. [31], this dataset includes 49 control and 20 HD postmortem BA9 brain samples.

RNA-Seq datasets were obtained from the NCBI GEO database, selecting samples from Brodmann area 9 (BA9) of the frontal cortex to minimize regional variability and ensure comparability across studies. Demographic and clinical metadata (age, sex, disease duration, postmortem interval) were extracted from the original GEO submissions and are summarized in Table 1. This standardized sampling strategy was adopted to reduce confounding effects arising from tissue heterogeneity and demographic imbalance.

To enhance comparability across studies, all datasets were derived from the Brodmann area 9 (BA9) of post-mortem frontal cortex tissue. According to the original GEO records, the cohorts comprised adults of both sexes (AD: 8 patients/9 controls, mean age ≈ 78 years; PD: 29 patients/44 controls, mean age ≈ 70 years; HD: 20 patients/49 controls, mean age ≈ 58 years). Only neurologically confirmed cases and matched controls were included. Although limited metadata were available for disease duration and post-mortem interval, analyses were restricted to datasets with comparable sequencing depth and Illumina HiSeq platforms to minimize batch and coverage effects.

### 2.2. Identification of Differentially Expressed Genes (DEGs)

Differential expression analysis identifies genes exhibiting statistically significant expression changes between disease and control samples. All analyses were performed in R (version 4.4.2) using the Bioconductor framework [32,33] to find DEGs in AD, PD and HD based on their linked controls. Firstly, we normalized the gene expression data employing the log2 transform and statistical techniques. To control rate of false discovery we used “Limma” package(version 3.54.0) from R programming language with Benjamini–Hochberg correction [24]. The important DEGs were determined using *p*-value less than 0.05 and a |logFC| > 1. This dual threshold ensured both statistical and biological relevance (a two-fold upregulation or 50% downregulation).

Overlapping DEGs across the three datasets (GSE53697, GSE68719, and GSE64810) were determined using the InteractiVenn tool (https://www.interactivenn.net/; accessed on 15 August 2024) [34] to visualize intersections.

(1) $$Common\;DEGs={DEGs}_{AD}\;\cap \;{DEGs}_{PD}\;\cap {DEGs}_{HD}$$

This integrative approach enabled the identification of genes that were consistently dysregulated across multiple neurodegenerative conditions.

### 2.3. Functional and Pathway Enrichment Analysis

To interpret the biological significance of the shared DEGs, functional enrichment [35] and pathway analysis were performed using Enrichr (https://maayanlab.cloud/Enrichr/ (accessed on 15 November 2024)) [36,37]. Enrichr computes enrichment based on Fisher’s exact test and a Z-score transformation to evaluate the overrepresentation of known gene sets. Enriched terms were ranked using a combined score integrating statistical significance and deviation magnitude:

Functional categories from Gene Ontology (GO) [38] were explored, covering Biological Process (BP), Molecular Function (MF), and Cellular Component (CC). Pathway enrichment [39] was assessed through KEGG, Reactome, and WikiPathways databases. A significance threshold of *p* < 0.05 was applied across all analyses.

### 2.4. Protein–Protein Interaction (PPI) Network Construction

To explore functional associations among shared DEGs, a PPI network [40] was constructed using STRING v12.0 (https://string-db.org/ (accessed on 10 January 2025)) [41] to reflect how our specified DEGs, as well as proteins, communicate physically and functionally with each other. A minimum confidence score of 0.15 was applied in STRING to maintain network connectivity given the small set of overlapping DEGs. The lowest score confidence criterion produced a sharp PPI network due to the small number of common DEGs. This relatively low cutoff was selected after testing higher thresholds, which resulted in fragmented networks and loss of biologically plausible interactions; therefore, the chosen value balanced inclusiveness and interpretability. The resulting network was visualized and analyzed in NetworkAnalyst; hub genes were identified as the nodes with the highest degree, representing central regulators in the network.

### 2.5. Protein Drug Interactions Assessment

Potential drug–target relationships were identified by integrating the shared DEGs with the DrugBank v5.0 database [42,43] through NetworkAnalyst [44] to conduct protein–drug interactions in order to find possible interactions between our common DEGs and medicines in the DrugBank dataset. High-degree drugs were prioritized as potential repurposing candidates targeting multiple neurodegenerative pathways.

### 2.6. Gene Disease Association Analysis

To explore systemic diseases and comorbidities linked to shared DEGs, a gene–disease association network was constructed using DisGeNET [45,46], which highlights the growing understanding of human genetic disorders. A network analyst was used to examine the gene–disease interaction to identify diseases and chronic complications correlated with common DEGs. These parameters identified the disease nodes most interconnected with mutual DEGs, providing insight into shared molecular etiologies and possible secondary complications.

## 3. Results

### 3.1. Identification of Differentially Expressed Genes (DEGs) and Common Signatures Among PD, HD, and AD

Human RNA-Seq datasets retrieved from the NCBI Gene Expression Omnibus (GEO) were analyzed to investigate shared transcriptomic alterations among Parkinson’s disease (PD), Huntington’s disease (HD), and Alzheimer’s disease (AD). Differentially expressed genes (DEGs) were identified using the criteria *p* < 0.05 and |log_2_ fold change (logFC)| > 1.

In the AD dataset (GSE53697), 262 DEGs were identified, including 95 upregulated and 167 downregulated genes. The HD dataset (GSE64810) yielded 1581 DEGs (722 upregulated, 859 downregulated), while the PD dataset (GSE68719) contained 537 DEGs (165 upregulated, 372 downregulated). Table 2 summarizes the datasets, including GEO accession numbers, tissue source, and DEG counts.

A comparative analysis using the InteractiVenn tool revealed 10 genes commonly dysregulated across all three disorders. Table 3 lists these shared DEGs, along with their respective logFC and *p*-values. All exhibited statistically significant differential expression (*p* < 0.05; |logFC| > 1) across the three diseases.

**Figure 2:**
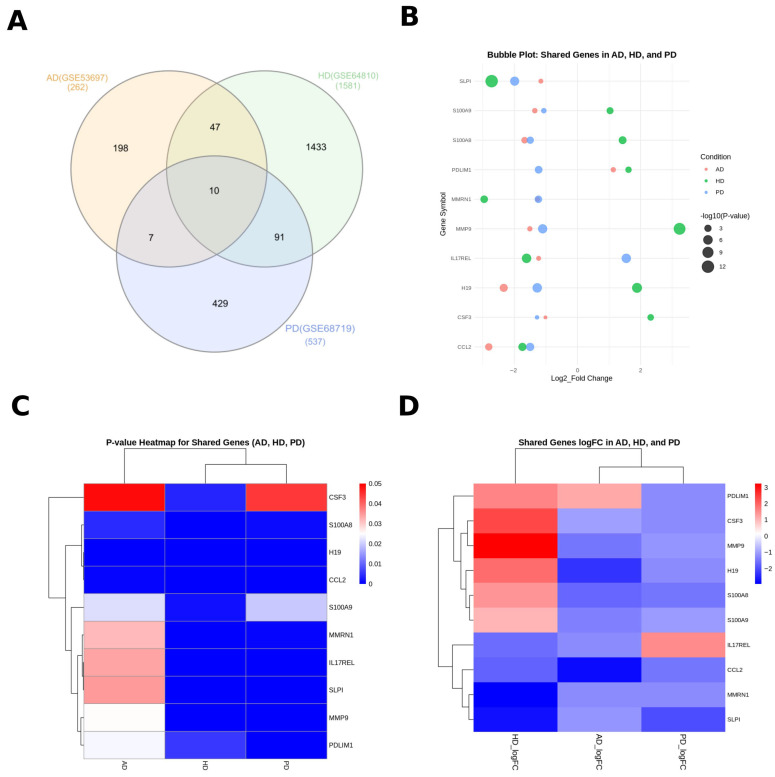
RNA-Seq analysis comparison between three diseases (PD, HD, and AD). (**A**) Venn diagram illustrating the number of differentially expressed genes (DEGs) shared among Parkinson’s disease (PD), Huntington’s disease (HD), and Alzheimer’s disease (AD). (**B**) Bubble plot depicting the joint log_2_ fold changes and corresponding *p*-values for the DEGs commonly identified across the three conditions. (**C**) Heatmap visualizing the statistical significance (*p*-values) of the shared DEGs across PD, HD, and AD. (**D**) Heatmap representing the log_2_ fold changes in the shared DEGs across the three disease datasets.

The Venn diagram (Figure 2A) illustrates the overlap among the three datasets, identifying 10 shared DEGs across AD, HD, and PD, 47 between AD and HD, 7 between AD and PD, and 91 between HD and PD. Figure 2B–D visualizes these shared genes through bubble and heat maps. Notably, *CCL2*, *MMP9*, and *S100A9* showed consistent dysregulation across all diseases with strong statistical significance. The integrated analysis highlights *MMP9*, *SLPI*, *CCL2*, and *S100A8/S100A9* as high-confidence pan-neurodegenerative biomarkers.

The ten shared DEGs exhibited heterogeneous expression directionality across the three disorders; some were upregulated in one condition and downregulated in another. This divergence likely reflects disease-specific compensatory or degenerative responses rather than analytical noise. Nevertheless, their consistent presence across all datasets suggests participation in convergent biological processes, particularly neuroinflammatory signaling, oxidative stress, and immune activation, which are well-recognized contributors to neurodegenerative progression.

In addition to these ten common DEGs, pairwise overlap analyses identified 47 shared genes between AD and HD, 7 between AD and PD, and 91 between HD and PD (Supplementary Tables S1–S3). These partial intersections highlight both shared and disease-specific transcriptional responses and provide complementary insight into the molecular crosstalk among these conditions.

In addition to the ten DEGs shared by all three disorders, pairwise overlaps were examined to capture partial convergence. Forty-seven genes were common to AD and HD, seven to AD and PD, and ninety-one to HD and PD. These intermediate overlaps reveal that HD shares a broader transcriptomic similarity with both AD and PD than do the latter two with each other.

Interestingly, several of the ten shared genes displayed opposite directions of regulation among diseases. For instance, *MMP9* and *S100A9* were up-regulated in HD but down-regulated in AD and PD. Such divergence may reflect disease-specific cellular contexts or compensatory immune activation states rather than experimental noise. Despite these differences, enrichment analysis indicated convergence at the pathway level, particularly in neuroinflammatory and cytokine-mediated signaling.

### 3.2. Functional and Pathway Enrichment Analysis

Gene Ontology (GO) and pathway enrichment analyses were performed using Enrichr to identify the biological functions and pathways associated with the shared DEGs. GO terms were categorized into Biological Process (BP), Molecular Function (MF), and Cellular Component (CC).

**Figure 3:**
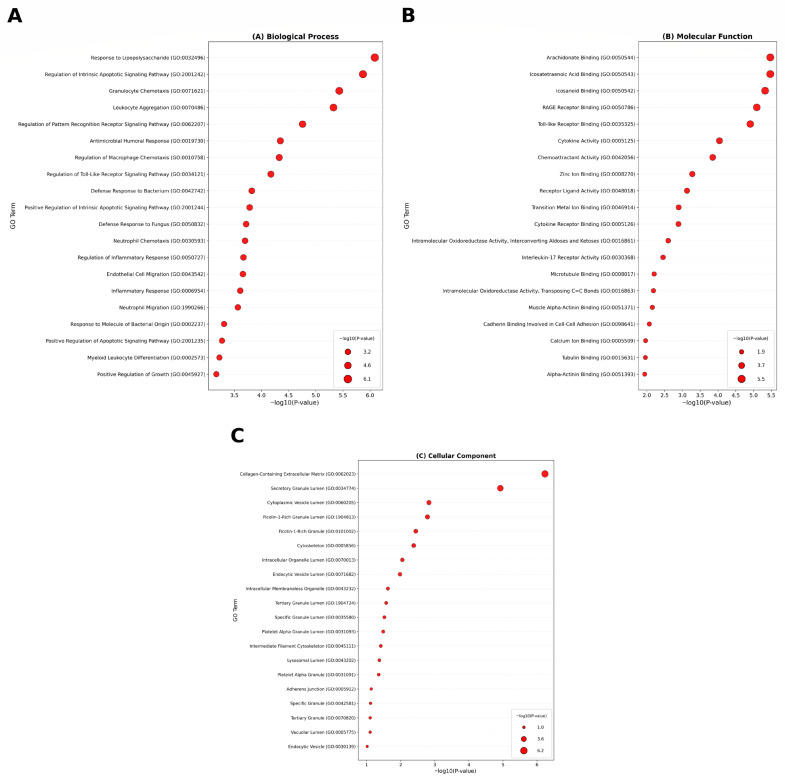
The bubble plot of gene ontology analysis of common DEGs between PD, HD, and AD conducted by Enrichr: (**A**) biological processes (BP), (**B**) molecular function (MF), and (**C**) cellular component (CC).

Significantly enriched BP terms included response to lipopolysaccharide, regulation of intrinsic apoptotic signaling pathway, granulocyte chemotaxis, and leukocyte aggregation. Enriched MF terms included arachidonate binding, icosanoid binding, RAGE receptor binding, and Toll-like receptor binding, while enriched CC terms were collagen-containing extracellular matrix, secretory granule lumen, and cytoplasmic vesicle lumen (Figure 3A–C).

These enrichments collectively indicate the central involvement of inflammatory and immune-related mechanisms in the shared pathology of AD, PD, and HD.

**Figure 4:**
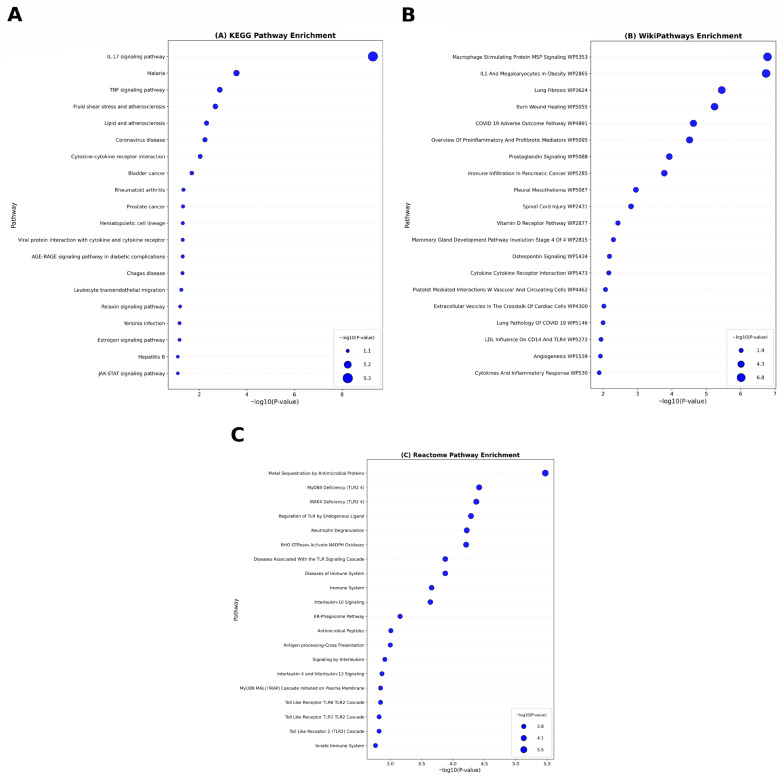
Pathway enrichment analysis bubble plot of common DEGs between PD, HD, and AD conducted by Enrichr: (**A**) KEGG pathway, (**B**) WikiPathway, and (**C**) Reactome pathway.

Pathway enrichment using KEGG, WikiPathways, and Reactome revealed significant activation of immune and inflammatory signaling cascades. Top pathways included the IL-17 signaling pathway, TNF signaling pathway, and neutrophil degranulation (Figure 4A–C), suggesting shared neuroimmune dysregulation as a key convergent mechanism across these diseases.

### 3.3. Protein–Protein Interactions Network and Identification of Hub Proteins

**Figure 5:**
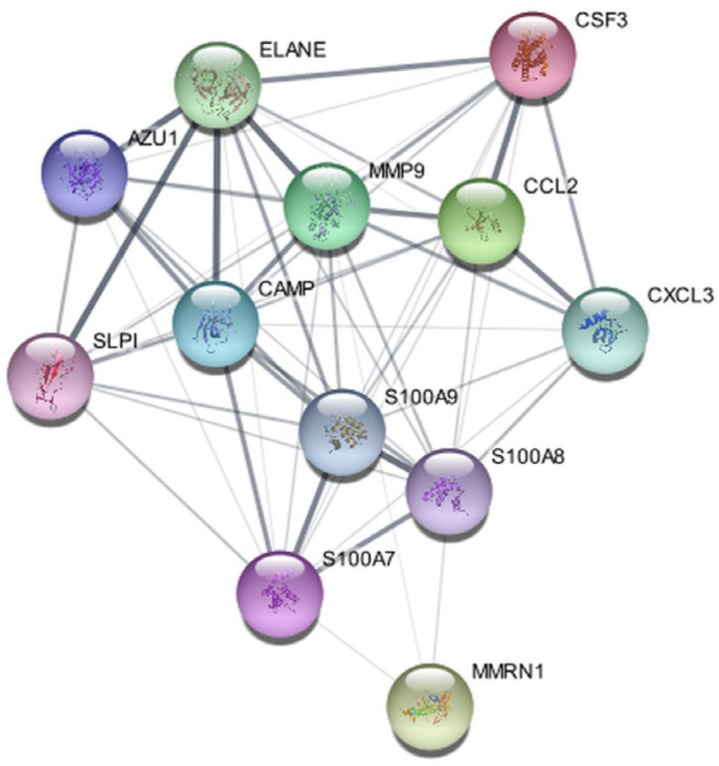
Protein–Protein Interactions Network of differentially expressed genes (DEGs) shared by PD, HD, and AD. The edges represent the interactions of the nodes and the circles of the nodes represent DEGs.

To elucidate functional interconnections among shared DEGs, a PPI network was constructed using STRING and visualized in Cytoscape. The resulting network (Figure 5) comprised 12 nodes and 56 edges, representing the molecular interaction landscape among common DEGs.

**Figure 6:**
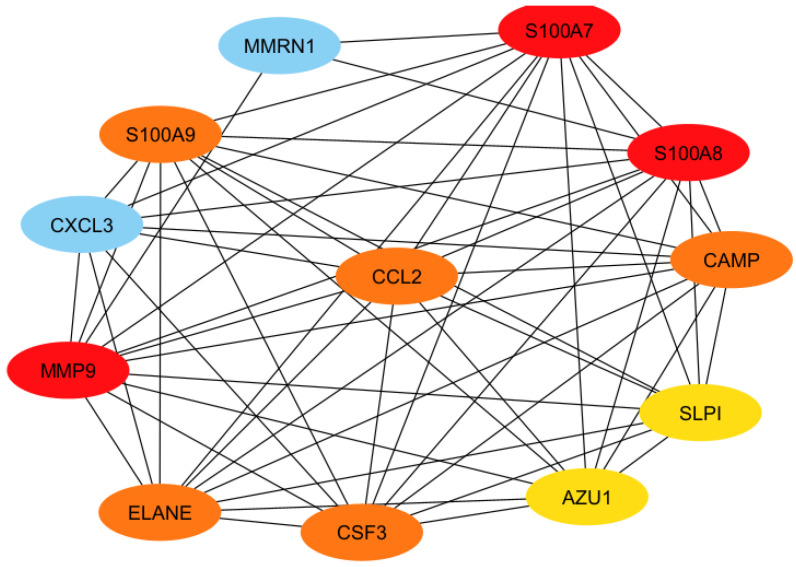
Hub genes identification from the PPI network using Cytohubba. The red nodes is the highest ranked and the blue nodes are the lowest ones.

Hub genes were identified using the Maximal Clique Centrality (MCC) algorithm in the CytoHubba plugin. The top ten hub genes—*S100A7*, *MMP9*, *S100A8*, *CAMP*, *ELANE*, *CCL2*, *S100A9*, *CSF3*, *AZU1*, and *SLPI*—were identified as the most functionally influential (Figure 6). These hub genes represent central molecular nodes potentially critical for the regulation of neuroinflammatory signaling in neurodegenerative disorders.

### 3.4. Identification of Candidate Therapeutic Compounds

**Figure 7:**
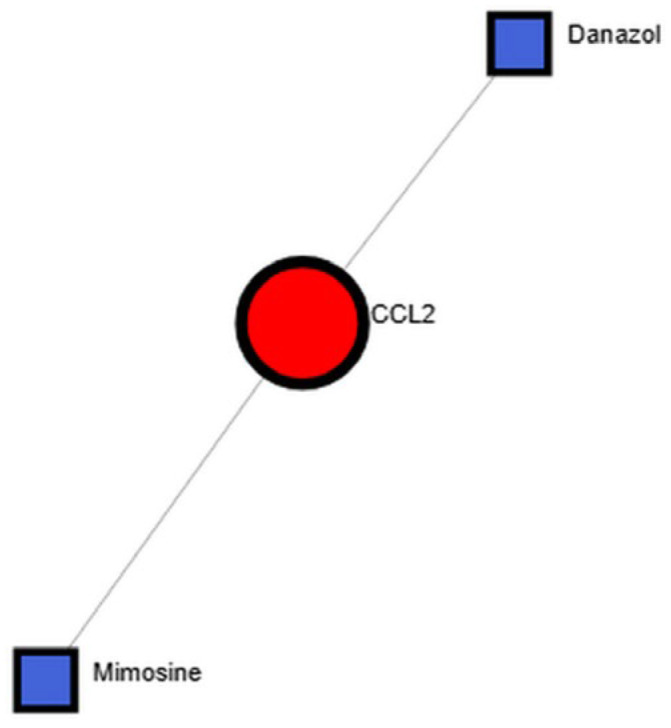
This network illustrates the interactions between candidate drugs and disease-associated proteins. A hub protein’s interconnection with its drugs is identified. The red circle is the hub gene and the square node is the drug.

Protein–drug interaction analysis using NetworkAnalyst identified potential compounds targeting hub proteins. As shown in Figure 7, the hub protein *CCL2* exhibited binding interactions with Danazol and Mimosine, two compounds previously reported to modulate immune and inflammatory pathways. These findings provide initial leads for potential therapeutic repurposing in AD, PD, and HD.

### 3.5. Validation and Functional Interpretation of Hub Genes

To strengthen the biological relevance of the identified hub genes, we cross-validated *MMP9*, *S100A8/A9*, *CCL2*, and *LCN2* using evidence from independent transcriptomic and proteomic datasets reported in the literature. These genes have been consistently implicated in neuroinflammatory signaling, glial activation, and synaptic dysfunction across Alzheimer’s, Parkinson’s, and Huntington’s diseases [47,48,49,50,51,52]. Previous multi-cohort meta-analyses and experimental studies confirmed that *MMP9* and *S100A8/A9* contribute to microglial activation and neuronal injury [47,48], while *CCL2* signaling has been linked to neurodegeneration and immune cell recruitment in Huntington’s disease [49]. *LCN2* has also emerged as a marker of neuroimmune response and oxidative stress [50]. Collectively, these reports reinforce the robustness of our findings and highlight that the identified hub genes participate in convergent inflammatory pathways underlying neurodegenerative processes [51,52].

### 3.6. Gene–Disease Association Analysis

**Figure 8:**
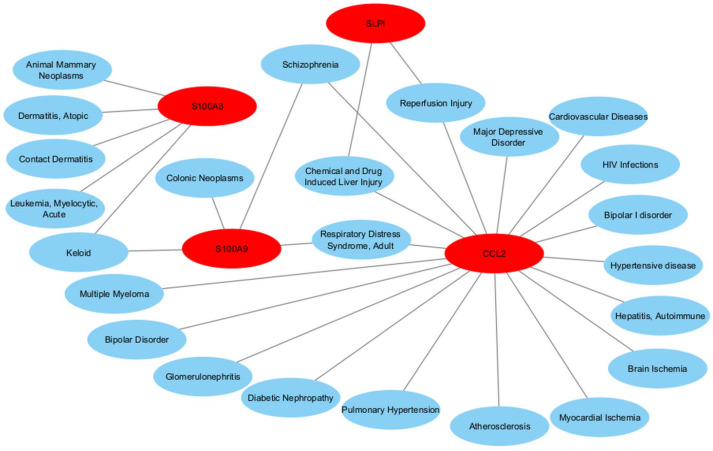
This network illustrates common DEG-related diseases. The blue ellipse node represents the disease and the red ellipse node represents the gene symbols.

Gene–disease association analysis was performed using NetworkAnalyst to examine potential links between identified hub genes and other human disorders. The analysis revealed strong associations with schizophrenia, bipolar disorder, unipolar depression, cardiovascular disease, and atherosclerosis (Figure 8). These relationships further support the systemic and cross-disease relevance of the identified genes, highlighting their potential as targets for multi-disease intervention strategies.

## 4. Discussion

In this research, a comparative transcriptomic analysis of postmortem brain RNA-Seq datasets from patients with Alzheimer’s disease (AD), Huntington’s disease (HD), and Parkinson’s disease (PD) was conducted. By integrating multiple bioinformatics pipelines, a set of shared differentially expressed genes (DEGs) were identified that may represent common molecular mechanisms underlying these three major neurodegenerative disorders. Such shared dysregulated genes have potential utility as diagnostic biomarkers and therapeutic targets.

The comparative analysis revealed ten genes—*H19*, *CCL2*, *CSF3*, *IL17REL*, *MMP9*, *PDLIM1*, *MMRN1*, *SLPI*, *S100A8*, and *S100A9*—that exhibited consistent differential expression patterns across AD, HD, and PD. These findings suggest a convergent transcriptional response contributing to overlapping pathological processes such as neuroinflammation, immune dysregulation, and neuronal stress responses. This supports the growing evidence that neurodegenerative diseases, despite having distinct etiologies, share common downstream mechanisms involving chronic inflammation and cellular stress.

Functional enrichment and pathway analyses provided further insights into the biological roles of these shared genes. Gene Ontology (GO) enrichment revealed significant enrichment in biological processes related to immune response, apoptosis regulation, and inflammatory signaling, while molecular function and cellular component categories implicated receptor binding, cytokine activity, and extracellular matrix organization. These enriched pathways are consistent with previous studies linking neuroinflammation and glial activation to neuronal loss in neurodegenerative disorders.

Pathway enrichment via KEGG, WikiPathways, and Reactome databases highlighted several key signaling cascades, including the IL-17 and TNF signaling pathways and neutrophil degranulation, indicating that innate immune activation is a shared hallmark across these diseases. Dysregulation of these pathways can drive microglial and astrocytic reactivity, leading to progressive neurodegeneration. The convergence of these inflammatory cascades provides a mechanistic rationale for considering anti-inflammatory interventions as cross-disease therapeutic strategies.

Protein–protein interaction (PPI) network analysis identified *S100A7*, *MMP9*, *S100A8*, *CAMP*, *ELANE*, *CCL2*, *S100A9*, *CSF3*, *AZU1*, and *SLPI* as hub genes, representing key molecular nodes that may coordinate these shared pathological networks. Many of these genes, such as *MMP9* and *CCL2*, have been previously implicated in blood–brain barrier disruption, leukocyte migration, and cytokine signaling in neurodegenerative disease models. Their centrality in the PPI network underscores their potential as molecular switches that integrate inflammatory and degenerative signaling.

Gene–disease association analysis revealed that these hub genes are also linked to other complex disorders, including schizophrenia, cardiovascular disease, bipolar disorder, and brain ischemia. Such cross-disease associations indicate that neurodegenerative gene networks exert systemic effects beyond the central nervous system and may share genetic and molecular susceptibilities with psychiatric and metabolic disorders.

Drug–protein interaction analysis further identified Danazol and Mimosine as potential compounds that interact with *CCL2*. Both compounds have reported immunomodulatory and anti-inflammatory properties, suggesting their potential as repurposable therapeutic candidates. However, further experimental and pharmacological validation is needed to confirm their efficacy and safety in neurodegenerative disease contexts.

Danazol, a synthetic steroid and weak androgen receptor agonist, has been reported to attenuate oxidative stress and neuroinflammatory signaling in neuronal models through androgen receptor-dependent mechanisms, thereby suggesting possible neuroprotective effects [53]. Mimosine, a naturally occurring iron-chelating amino acid, exhibits antioxidative and mitochondrial-protective properties that may counteract iron-induced oxidative stress and neuronal injury [54]. Although these findings are preliminary and based primarily on in vitro or animal model data, they provide a rationale for exploring drug-repurposing strategies in neurodegenerative disorders. Integrating transcriptomic signatures with pharmacologic data may thus accelerate the identification of candidate therapeutics with shared mechanisms of action across multiple neurodegenerative diseases [55,56].

Collectively, our findings support the hypothesis that AD, PD, and HD share overlapping molecular signatures driven by immune and inflammatory dysregulation. The identified hub genes and pathways not only enhance understanding of shared neurodegenerative mechanisms but also provide a foundation for the development of multi-target or repurposed therapeutic strategies aimed at modulating these common pathways.

Our results highlight a shared neuroinflammatory signature across Alzheimer’s disease (AD), Parkinson’s disease (PD), and Huntington’s disease (HD), characterized by the dysregulation of hub genes such as *MMP9*, *S100A8*, *S100A9*, *CCL2*, and *LCN2*. Independent transcriptomic and proteomic analyses have consistently implicated these genes in glial activation, cytokine signaling, and blood–brain barrier dysfunction [47,48,49,50,51,52]. For example, *MMP9* and *S100A8/A9* are strongly associated with microglial activation and synaptic remodeling [47,48], *CCL2* mediates immune-cell recruitment and neuroinflammation in HD and AD models [49], and *LCN2* serves as an astrocytic marker of oxidative stress and neuronal injury [50]. These findings are supported by multi-cohort integrative studies linking inflammatory mediators to cortical atrophy, cerebrospinal-fluid biomarkers, and cognitive decline in longitudinal neurodegeneration cohorts [55,56]. Together, these convergent data confirm the biological plausibility of the hub genes identified in our comparative analysis and suggest that inflammatory and immune-response pathways represent common therapeutic axes across neurodegenerative disorders [51,52].

Several limitations should be acknowledged. First, the sample sizes—particularly for AD—were relatively small, which may limit generalizability. Second, inherent heterogeneity among datasets (postmortem interval, age, and RNA integrity) could introduce bias despite normalization. Third, the inclusion of Huntington’s disease, a monogenic disorder, alongside polygenic diseases may influence comparative outcomes. Finally, validation using independent cohorts or experimental techniques such as qPCR or immunohistochemistry would strengthen the robustness of the findings. Future research integrating larger and multimodal datasets is warranted.

## 5. Conclusions

In this research, an integrative bioinformatics approach was employed to identify common molecular signatures and potential biomarkers shared among Alzheimer’s disease (AD), Parkinson’s disease (PD), and Huntington’s disease (HD). By overlapping and filtering gene expression profiles from independent RNA-Seq datasets, we identified shared differentially expressed genes and constructed their associated Gene Ontology (GO) terms, signaling pathways, and protein–protein interaction (PPI) networks. The analysis further revealed potential protein–drug associations, providing insight into candidate compounds that may modulate disease-relevant targets.

These findings offer a systems-level perspective of the convergent molecular mechanisms driving neurodegeneration, emphasizing the critical roles of immune and inflammatory pathways. The identified hub genes—particularly *MMP9*, *CCL2*, and *S100A8/A9*—may serve as promising candidates for therapeutic intervention or biomarker development across multiple neurodegenerative diseases. Moreover, the study underscores the translational potential of network-based analyses in informing personalized medicine strategies.

Nevertheless, certain limitations must be acknowledged. The research relied on publicly available RNA-Seq datasets, some of which had relatively small sample sizes that may limit statistical power and generalizability. Additionally, the analyses were computational and predictive; therefore, experimental validation through in vitro and in vivo models is essential to confirm the biological relevance of these findings. Future research incorporating larger, clinically diverse cohorts and integrating multi-omics data (e.g., proteomics, metabolomics, and epigenomics) will further refine our understanding of shared neurodegenerative mechanisms and accelerate the identification of actionable therapeutic targets.

## Figures and Tables

**Table 1:** Overview of analyzed RNA-Seq datasets showing the number of control and disease samples from BA9 brain tissue.

Disease Name	GEO Accession	Tissue Source	Normal Samples	Patient Samples	Total Samples
Parkinson’s Disease (PD)	GSE68719 [18]	postmortem human brain (BA9)	44	29	73
Huntington’s Disease (HD)	GSE64810 [19]	postmortem human brain (BA9)	49	20	69
Alzheimer’s Disease (AD)	GSE53697 [20]	postmortem human brain (BA9)	9	8	17

**Table 2:** Summary of analyzed RNA-Seq datasets and corresponding DEGs.

Disease Name	GEO Accession ID	Brain Tissue Source	Number of Total DEGs	Number of Up-Regulated DEGs	Number of Down-Regulated DEGs
Parkinson’s Disease (PD)	GSE68719 [18]	postmortem human (BA9)	537	165	372
Huntington’s Disease (HD)	GSE64810 [19]	postmortem human (BA9)	1581	722	859
Alzheimer’s Disease (AD)	GSE53697 [20]	postmortem human (BA9)	262	95	167

**Table 3:** Common DEGs shared by PD, HD, and AD.

Gene Symbol	logFC of AD	*p*-Value of AD	LogFC of HD	*p*-Value of HD	LogFC of PD	*p*-Value of PD
*H19*	−2.335	1.20 × 10^−4^	1.887	1.93 × 10^−7^	−1.2741803	1.08 × 10^−6^
*CCL2*	−2.81	8.75 × 10^−4^	−1.743	9.45 × 10^−5^	−1.4988129	8.06 × 10^−5^
*CSF3*	−1.013	4.87 × 10^−2^	2.319	3.79 × 10^−3^	−1.28162	4.45 × 10^−2^
*IL17REL*	−1.23	3.36 × 10^−2^	−1.612	1.30 × 10^−6^	1.5461572	1.49 × 10^−6^
*MMP9*	−1.508	2.53 × 10^−2^	3.235	3.01 × 10^−11^	−1.1023493	2.68 × 10^−6^
*PDLIM1*	1.135	2.43 × 10^−2^	1.617	5.53 × 10^−3^	−1.2294081	3.55 × 10^−4^
*MMRN1*	−1.262	3.16 × 10^−2^	−2.954	4.68 × 10^−4^	−1.2365235	5.85 × 10^−4^
*SLPI*	−1.159	3.47 × 10^−2^	−2.718	1.00 × 10^−12^	−1.9889283	4.52 × 10^−6^
*S100A8*	−1.674	4.20 × 10^−3^	1.43	2.07 × 10^−4^	−1.49514	1.02 × 10^−3^
*S100A9*	−1.351	2.19 × 10^−2^	1.032	1.79 × 10^−3^	−1.0651068	2.00 × 10^−2^

## Data Availability

Data derived from public domain resources. The data presented in this study are available in the NCBI Gene Expression Omnibus (GEO) repository at https://www.ncbi.nlm.nih.gov/geo/ (accessed on 25 July 2024), reference numbers GSE68719, GSE64810 and GSE53697. These data were derived from the following resources available in the public domain: Parkinson’s Disease (PD): GSE68719 [18], Huntington’s Disease (HD): GSE64810 [19], Alzheimer’s Disease (AD): GSE53697 [20].

## Supplementary Materials

The supporting information can be downloaded at: https://www.mdpi.com/article/10.3390/cimb47120976/s1, Table S1: DEGS for AD; Table S2: DEGS for HD; Table S3: DEGS for PD.
